# Highly reliable, targeted photothermal cancer therapy combined with thermal dosimetry using a near-infrared absorbent

**DOI:** 10.1038/s41598-020-66646-x

**Published:** 2020-06-17

**Authors:** Shinsuke Nomura, Yuji Morimoto, Hironori Tsujimoto, Masashi Arake, Manabu Harada, Daizoh Saitoh, Isao Hara, Eiichi Ozeki, Ayano Satoh, Eiji Takayama, Kazuo Hase, Yoji Kishi, Hideki Ueno

**Affiliations:** 1 Department of Surgery, National Defense Medical College, Saitama, 359-8513 Japan; 2 Department of Physiology, National Defense Medical College, Saitama, 359-8513 Japan; 3 Division of Traumatology, National Defense Medical College Research Institute, Saitama, 359-8513 Japan; 4 Technology Research Laboratory, Shimadzu Corporation, Kyoto, 604-8511 Japan; 5 Graduate School of Interdisciplinary Science and Engineering in Health Systems, Okayama University, Okayama, 700-0082 Japan; 6 Department of Oral Biochemistry, Asahi University School of Dentistry, Gifu, 501-0296 Japan

**Keywords:** Biophysics, Biotechnology, Cancer, Oncology

## Abstract

Photothermal therapy (PTT) using a photo-absorbent in the near-infrared (NIR) region is an effective methodology for local cancer treatment. Before PTT using a NIR absorbent is executed, the operator generally determines the two parameters of fluence rate and irradiation time. However, even if the irradiation parameters are unchanged, the therapeutic effect of PTT is often different for individual tumors. Hence, we examined the therapeutic effect of PTT using a NIR absorbent (ICG lactosome) while changing two parameters (fluence rate and irradiation time) in various combinations. As a result, there was no robust correlation between those parameters and the therapeutic effect. Compared to those parameters, we found that a more reliable determinant was maintenance of the tumor temperature above 43 °C during NIR irradiation. To reconfirm the significance of the determinant, we developed a new system that can regulate the temperature at the NIR irradiation site at a constant level. By using the new system, we verified the treatment outcomes for tumors in which the NIR absorbent had accumulated. All of the tumors that had been kept at 43 °C during NIR irradiation were cured, while none of the tumors that had been kept at a temperature below 41 °C were cured. In conclusion, PTT using a NIR absorbent with thermal dosimetry is a highly reliable treatment for cancer.

## Introduction

Photothermal therapy (PTT) is a cancer treatment that induces cancer cell death by heat generated in tumor tissue exposed to near-infrared (NIR) light^1^. NIR absorbents are used for facilitating efficient heat production^2^. Methods for enhancing selective accumulation in a lesion by incorporating a drug delivery system (DDS) into a NIR absorbent have recently been investigated^3^. The introduction of a DDS results in improvement in the efficiency of heat production in tumor tissue while minimizing photothermal damage to surrounding normal tissue and may lead to reliable PTT with high efficiency and excellent safety.

When executing PTT using a DDS-type NIR absorbent, the tumor is irradiated with a NIR light at a certain time after administration of the absorbent. Irradiation parameters such as fluence rate and irradiation time in PTT are generally determined prior to NIR irradiation. However, since it is almost impossible to obtain accurate tumor-related information (tumor size, heterogeneity of the tumor tissue, and distribution of NIR absorbents inside the tumor)^4,5^ before PTT, it is difficult to exert a maximum therapeutic effect with preset irradiation parameters. Therefore, irradiation conditions that increase the fluence rate or lengthen the irradiation time have been used to enhance the anti-tumor effect of PTT^6,7^. In other words, since it is not easy to perform NIR irradiation with optimized parameters depending on each individual tumor, a strategy of setting an excessive dose of irradiation has been taken to avoid a poor outcome due to an insufficient dose of irradiation. However, in cases in which such a strategy is used, the tumor tissue is often heated to 50 °C or higher, and damage to surrounding normal tissues can easily occur. A NIR laser with an excessive fluence rate has been reported to cause collateral thermal damage, such as burn, inflammation and edema, to tissue surrounding the tumor^8,9^. When tissue is heated to a temperature of 60 °C or higher, coagulative necrosis inevitably occurs since most proteins are denatured at 60 °C.

We therefore pursued the optimal methodology for increasing the probability of tumor cure without heating the tumor tissue more than necessary when performing PTT using DDS-type NIR absorbents. As the first step for the pursuit of an optimal method, we studied the outcomes of PTT using a DDS-type NIR absorbent for cancer tumors (cure/growth) when irradiation parameters (fluence rate and irradiation time) were changed. In addition, the temperature of the tumor during NIR irradiation was measured, and correlations between the highest temperature reached during NIR irradiation and treatment outcomes were also verified. As a result, we found a determinant for improving the probability of tumor cure without an excessive dose of irradiation. Furthermore, we developed a new PTT treatment system of which the working principle fulfills the determinant, and we obtained complete regression of tumors by using the system.

## Results

### Outcomes of PTT using a DDS-type NIR absorbent when changing irradiation parameters

The effects of irradiation parameters (fluence rate and irradiation time) on tumors (cure/growth) after PTT using a DDS-type NIR absorbent (ICG lactosome) were examined.

Forty-eight hours after administration (i.v.) of a DDS-type NIR absorbent to tumor-bearing mice (8.8 mg/kg), the tumors were irradiated using a NIR laser (808 nm) with fluence rates of 250, 500, 750 and 1000 mW/cm^2^ and with irradiation times of 111, 222, 333, 666 and 1000 s.

**Figure 1:**
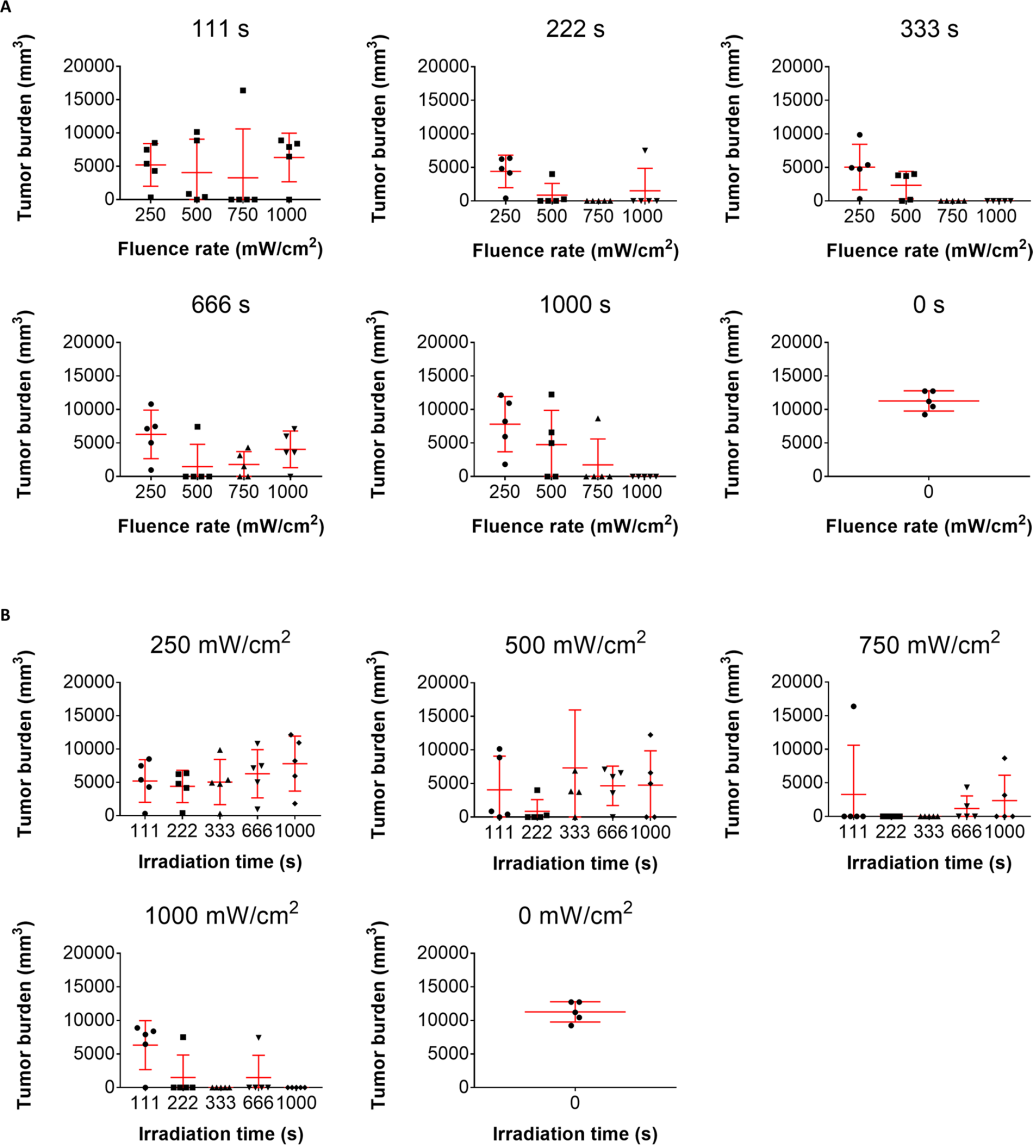
(**A**) Scatter plots of tumor burdens in mice when irradiated at a fluence rate of 250, 500, 750, 1000 or 0 mW/cm^2^. Data were obtained on the 21st day after irradiation (n = 5 each). (**B**) Scatter plots of tumor burdens in mice when irradiated with irradiation time of 111, 222, 333, 666, 1000 or 0 s. Data were obtained on the 21st day after irradiation (n = 5 each).

Tumor sizes at 21 days after irradiation are shown in Fig. 1. In each graph in Fig.1A, the tumor size is plotted as a function of fluence rate at a constant irradiation time. Negative correlations between fluence rate and tumor size were seen at irradiation times of 333 s (r = −0.715, P < 0.0004) and 1000 s (r = −0.653, P < 0.0018), while there was no significant correlation between fluence rate and tumor size at irradiation times of 111, 222 and 666 s (Fig. 1A). When tumor size was plotted as a function of irradiation time at a constant fluence rate (Fig.1B), a significant correlation between irradiation time and tumor size was found only for the irradiation time of 1000 s (r = −0.428, P < 0.033).

All of the mice treated with irradiation for 333 s and 1000 s at 1000 mW/cm^2^ and with irradiation for 222 s and 333 s at 750 mW/cm^2^ showed tumor eradication. Based on the results, the optimal irradiation parameter setting that is most likely to eradicate tumors is 1000 s at 1000 mW/cm^2^. That is, as described in the introduction section, excessive irradiation is needed when aiming for complete tumor eradication. However, tumor eradication was also seen some mice treated with a fluence rate of <1000 mW/cm^2^ or irradiation time of <1000 s (e.g., four out of 5 tumors were eradicated for 666 s at 500 mW/cm^2^). Accordingly, fluence rate and irradiation time may not be pertinent determinants of tumor eradication. Hence, we focused on the temperature during PTT.

### Effect of temperature during PTT using a DDS-type NIR absorbent on treatment outcomes

We also investigated the effect of temperature of the tumor during PTT on treatments outcomes (cure/growth).

**Figure 2:**
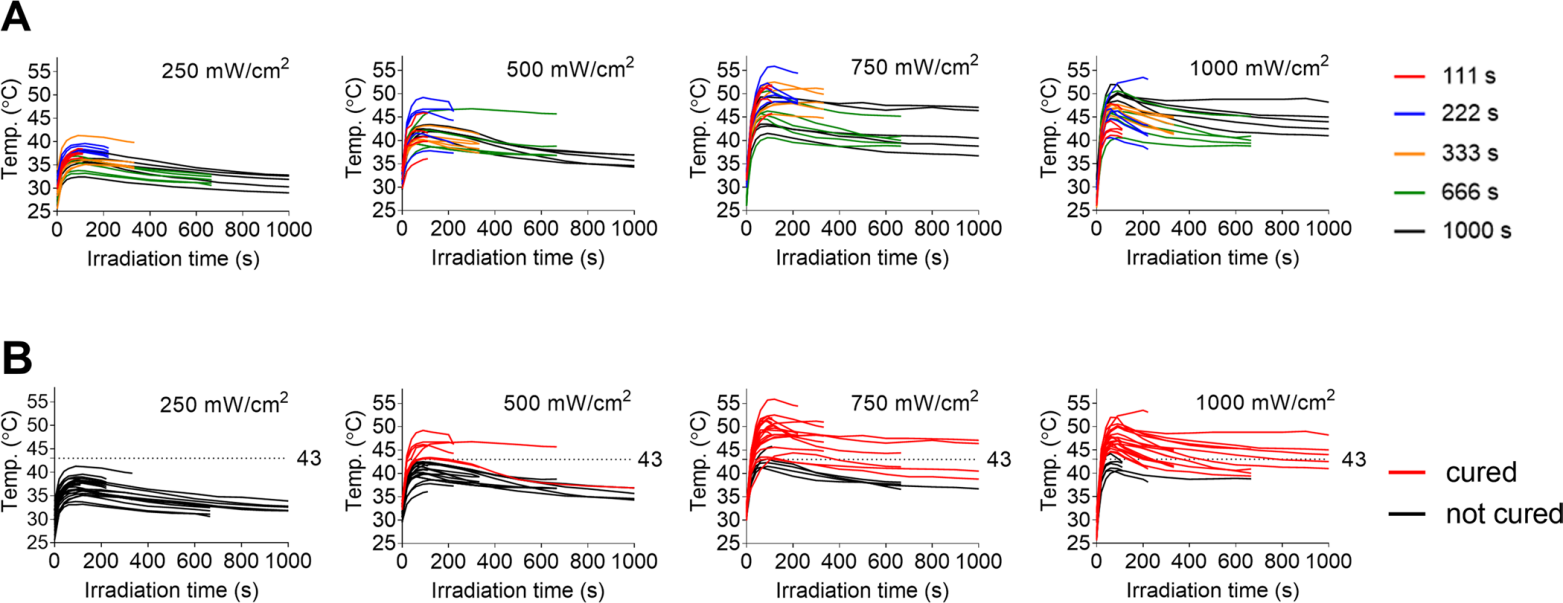
(**A**) Raw profile of temperature change in each tumor during NIR irradiation. (**B**) The temperature curves in “A” are color-coded into two groups. The tumors for which the temperature curves are shown in red were eradicated (cured) and the tumors for which the temperature curves are shown in black were not cured (proliferation of tumor cells).

When each mouse was being irradiated with the NIR laser, the temperature at the surface of the skin covering the tumor was recorded. Figure 2A shows the time course of temperature at each fluence rate. In the time courses at all fluence rates, the temperature reached a peak at 100–120 s after the start of NIR irradiation and then remained constant or gradually decreased. A comparison of the four graphs indicates that the temperature during NIR irradiation tended to increase with an increase in fluence rate.

**Figure 3:**
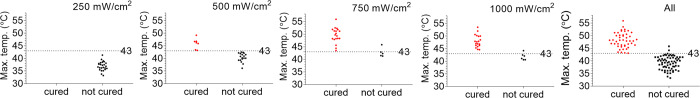
Scatter plots of the maximum temperature in each tumor during NIR irradiation. Each plot is divided into two groups (“cured” and “not cured”). Fluence rate (mW/cm^2^) is shown in each graph. The results indicate that all but two of the 49 tumors were cured at a temperature above 43 °C regardless of the fluence rate: the two cases of “not cured” tumor were irradiated for 111 s.

However, as indicated by the relationship between the time course of temperature and treatment outcome (Fig. 2B), the tumors disappeared when the temperature of the tumors increased above 43 °C (Fig. 2B): all but two of the 49 tumors disappeared regardless of the fluence rate (Fig. 3). The two tumors that did not disappear were included in the groups of 750 and 1000 mW/cm^2^ (Fig. 3) and both tumors were irradiated for 111 s. The results suggested that tumor cure depends on the tumor temperature during NIR irradiation for more than 111 s.

### Verification of the antitumor effect of temperature in PTT using a DDS-type NIR absorbent

For verification of the antitumor effect of temperature during PTT using a DDS-type NIR absorbent, we developed a thermal sensor circuit-based NIR laser irradiation system using a non-contact thermometer (FT- H10; Keyence) in order to keep the temperature of the irradiated target constant during irradiation^10^. We carried out a NIR irradiation experiment using the temperature-feedback laser system.

Forty-eight hours after administration (i.v.) of ICG lactosome to tumor-bearing mice (8.8 mg/kg), tumors were irradiated at 40, 41, 42 or 43 °C for 333 s using the system and tumor size was measured up to 21 days after irradiation. The results showed that the tumor was not cured at a temperature below 41°C. However, 3 of 5 tumors were cured at 42°C and all of the tumors were cured at 43 °C (Table 1). Similar results were obtained in *in vitro* experiments in which tumor cell viability declined steeply from 42 °C to 43 °C (Fig. S1).

**Table 1:** Treatment outcomes with the temperature-controlled NIR laser irradiation system in intradermal tumor model mice administered ICG lactosome (8.8 mg/kg) at 48 h before the laser irradiation.

Preset temperature (°C)	Measured temperature (means ± SD)	Outcome (Cured tumors/All tumors)
40	40.19 ± 0.81	0/5
41	41.21 ± 0.83	0/5
42	42.26 ± 0.85	3/5
43	43.13 ± 0.79	5/5

Temperature was preset at 40, 41, 42, or 43 °C (n = 5 each). The “measured temperature” was the value just before the end of irradiation. The thermal measurement was at a refresh rate of 10/s.

### Negligible increase in temperature at the irradiated non-tumor site

**Figure 4:**
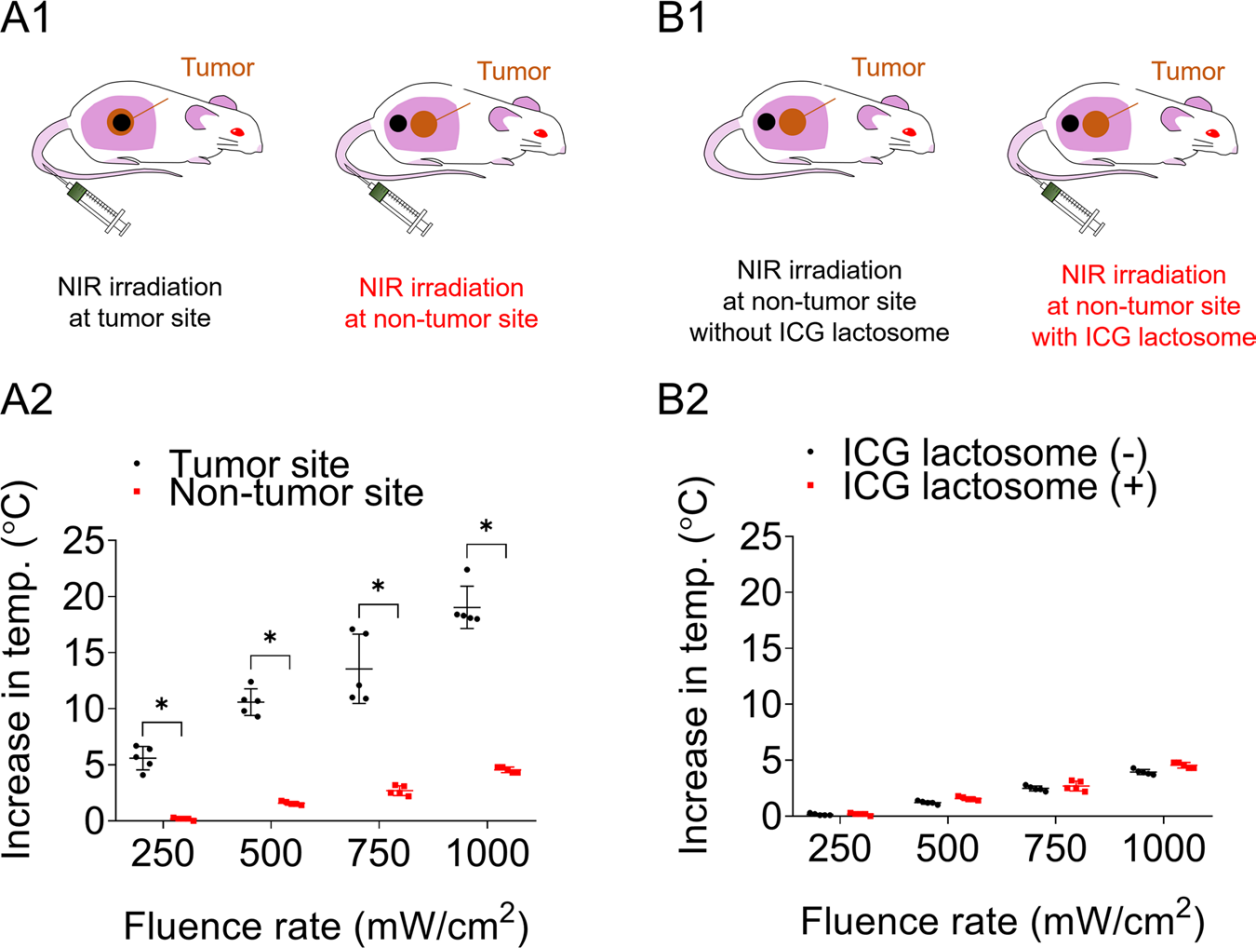
Increase in temperature at the spot of NIR irradiation on the tumor site or at that on a non-tumor site in mice with ICG lactosome administration (A1,A2). A1 Schema of the NIR irradiation point (black circle); A2 Scatter plots of increase in temperature at the tumor site and non-tumor site with ICG lactosome administration. With ICG lactosome administration, the higher the fluence rate was, the greater was the difference in increases in temperature at the tumor site and non-tumor site (*P < 0.01, n = 5 each). Increase in temperature at the spot of NIR irradiation on a non-tumor site in mice with or without ICG lactosome administration (B1,B2). B1 Schema of the NIR irradiation point (black circle); B2 Scatter plots of increase in temperature at the non-tumor site with and without ICG lactosome administration. Increase in temperature at the non-tumor site with ICG lactosome administration is hardly distinguishable from that without ICG lactosome administration.

To confirm that the NIR absorbent does not accumulate in tissue other than the tumor tissue and that the increase in temperature at non-tumor sites is negligible, temperatures of the tumor site and non-tumor site were measured during NIR laser irradiation at 48 hours after the injection of ICG lactosome (8.8 mg/kg) (Fig. 4A1). The increases in temperature (means ± SD) at the tumor site from the initial temperature to maximum temperature at fluence rate of 250, 500, 750 and 1000 mW/cm^2^ were 5.6 ± 1.0, 10.6 ± 1.2, 13.6 ± 3.1 and 19.0 ± 1.9 °C, respectively, while those at the non-tumor site were 0.2 ± 0.1, 1.6 ± 0.2, 2.7 ± 0.4 and 4.6 ± 0.3 °C, respectively (Fig. 4A2): the higher the fluence rate was, the larger was the difference in temperature increase.

We also compared the differences in temperature increases at non-tumor sites with and without injection of the NIR absorbent (Fig. 4B1). In the case of ICG lactosome injection, the non-tumor site was irradiated at 48 hours after the injection. A slight increase in temperature during NIR irradiation was observed, and the increase in temperature was correlated with increase in fluence rate (Fig. 4B2). However, the increase in temperature at the non-tumor site in the case of NIR absorbent injection was almost same as that at the non-tumor site in the case of no NIR absorbent injection (Fig. 4B2). These results suggest that there is little accumulation of the NIR absorbent at sites other than the tumor site.

## Discussion

This study on PTT with a NIR absorbent (ICG lactosome) showed that tumors were cured when the maximum temperature of the tumor reached and exceeded 43 °C during NIR irradiation. It was also found that fluence rate and irradiation time were not reliable determinants for tumor cure compared to raising the temperature over 43 °C.

The results suggest that thermal dosimetry during NIR irradiation is a more reliable determinant than fluence rate or irradiation time for prediction of the therapeutic effect of PTT using a NIR absorbent. We verified this concept by using a temperature-controlled NIR laser system that can alter the output power of the laser to keep the tumor surface temperature constant. A temperature of 43 °C during PTT using the NIR absorbent was revealed to be critical for cure of an irradiated tumor.

Generally, the protocols of phototherapies are set before laser irradiation despite of the difference of the tumor size, the heterogeneity of cancer tissue^11^, the abnormal structure of tumor blood vessels^12^ and the tumor interstitial pressure^13^. Therefore, it is difficult to completely eradicate cancer in an actual clinical setting. Temperature-controlled PTT with a NIR absorbent can overcome this problem for any tumor status, and a highly reliable therapeutic effect can be achieved. Thermal dosimetry-assisted PTT with a NIR absorbent is useful clinically not only because it can effectively cure tumors but also because it does not damage normal tissues.

There are two limitations in this study. First, the tumor model was an intradermal tumor model for monitoring the temperature of skin above the tumor by using a non-contact thermometer. The exact temperature inside the tumor was not known since it was not measured in this experiment. However, the surface temperature of the tumor has been shown to be correlated with the internal temperature in an intradermal tumor model^14^. To confirm the correlation, we tried to measure the temperature inside the tumor using an ultrathin (diameter of 0.2 mm) thermocouple. However, it was too difficult to insert the thermocouple accurately into the target position in tumors because the needle was easily bent due to the hardness of the tumor and instability of the insertion angle of the thermocouple and we were not able to place the ultrathin thermocouple inside the tumor with accuracy at the millimeter level. As a result, reliable data for temperature inside the tumor were not obtained. However, Jacques *et al*. reported that existence of a NIR absorbent (ICG) in a tissue phantom increases the temperature inside the phantom: the experimental setting of a chicken tissue slab with embedded ICG gel using a NIR laser (805 nm) showed that the temperature at a depth of 11.3 mm was higher than that at a depth of 6.0 mm^15^. Considering the thermal effect of a NIR absorbent, even in our experimental conditions, the tumor internal temperature might have been almost the same as or even higher than the surface temperature. The other limitation in this study is that only colon cancer was examined. The tissue structure, heat tolerance and therapeutic threshold temperature might be different in other cancer types.

## Conclusions

We studied what are the reliable determinant in the therapeutic effect of photothermal therapy (PTT) using ICG lactosome, which is a DDS-type NIR absorbent. When the surface temperature of the tumor exceeded 43 °C during PTT with an irradiation time of >111 s, the tumor disappeared regardless of the fluence rate. Therefore, thermal dosimetry during PTT using a NIR absorbent is a highly reliable method for complete cancer eradication and will provide clinicians with a more reliable cancer treatment.

## Materials and Methods

### Tumor-targeted NIR absorbent

Indocyanine green (ICG) lactosome (ICG lactosome), which we previously developed, was used as a DDS-type NIR absorbent^16^ showing a tumor-specific uptake maximum at 48 h after systemic injection (Supplementary Information 2–4). In addition, it has already been shown in a gastric cancer model that ICG lactosome has characteristics of tumor tissue-specific accumulation such as peritoneal dissemination^17^ and metastatic lymph nodes^18^.

ICG lactosome is a micelle-based agent that was synthesized as previously reported^17,18^. ICG lactosome is an aggregate of two types of amphiphilic polymers, poly (sarcosine)–poly (L-lactic acid) (PS-PLLA) and the other type is ICG– poly (L-lactic acid) (ICG-PLLA). The composition ratio is approximately 80% of PS-PLLA and 20% of ICG-PLLA^17^.

Next, how to load the ICG onto the ICG lactosome is briefly explained. For ICG-PLLA synthesis (Fig. S5), the terminal end of PLLA was chemically modified using ICG as follows: 1.0 mg ICG–OSu was added to a dimethylformamide (DMF) solution of the free amino group bearing PLLA (2.46 mg), which contains an amino group designed as an indicator of sarcosine N-carboxyanhydride (NCA) polymerization during the synthesis of amphiphilic PS-PLLA-block copolymers. The reaction mixture was stirred at room temperature overnight under a light-shielding condition. Then the reaction mixture was purified by size-exclusion chromatography using a Sephadex LH–20 column (GE Healthcare Japan Corporation, Tokyo, Japan) with DMF as the eluent. ICG-PLLA was encapsulated in a polymeric micelle by blending with PS-PLLA in a thin film using the film rehydration technique. The solvent was removed under reduced pressure and the thin film that had formed was dissolved in ultrapure water. The resulting aqueous solution was filtered through a filter with a pore size of 0.2 µm and then freeze-dried to obtain ICG lactosome^17,18^.

The zeta potential of ICG lactosome was shown to be −0.51 mV in our previous study^17^. Detailed properties of ICG lactosome are given in Supplementary Information 6–8.

### Animals

Female Balb/c mice at 6 weeks of age (Japan SLC, Hamamatsu, Japan) were fed under specific pathogen-free conditions. All animal procedures followed the guidelines approved by the National Defense Medical College Animal Care and Use Committee.

### Cell lines

A murine Colon26 cell line (kindly supplied by the National Cancer Center (Tokyo, Japan)) stably expressing Nano-lantern^19^ (NLC26) was established as previously described^20^. The cells were cultured in DMEM medium (Sigma-Aldrich, St. Louis, MO) supplemented with 10% heat-inactivated fetal bovine serum (Life Technologies, Carlsbad, CA), 100 U/mL of penicillin, 100 µg/mL of streptomycin and 0.25 µg/mL of amphotericin B (Antibiotic-Antimycotic, Life Technologies) at 37 °C in 5% CO2 with 95% humidity.

### Establishment of a mouse intradermal tumor model

To establish the experimental intradermal tumor model, mice were injected with 0.5 × 10^6^ NLC26 cells suspended in 50 µL of phosphate-buffered saline into the skin of the right back under anesthesia. A combined anesthetic, prepared with 0.3 mg/kg of medetomidine, 4.0 mg/kg of midazolam and 5.0 mg/kg of butorphanol, was used^21^.

### NIR laser irradiation and thermal dosimetry

ICG lactosome was intravenously administered 4 or 5 days after intradermal inoculation of the cancer cells. At that time, the tumor of the right back was evident because of the cancer cell growth, and the size of the tumor was approximately 200 mm^3^. After taking the *in vivo* images, the tumor was irradiated using a fiber-coupled laser system with a laser diode at 808 nm (model FC-W-808, maximum output: 10 W; Changchun New Industries Optoelectronics Technology Co., Ltd., Jilin, China)^17^. The fiber probe was placed just above the tumor so that the irradiated light spot was 1.0 cm in diameter, corresponding to a spot area of 0.79 cm^2^. The fluence rate was set at 250, 500, 750 or 1000 mW/cm^2^ and the irradiation time was set at 111, 222, 333, 666 or 1000 s. The temperature of the tumor surface was measured with an infrared radiation thermometer (FT- H10; Keyence). Tumor size was repeatedly measured using a digital caliper until the 21st day after irradiation, and tumor volume was calculated by the following equation: (longitudinal size) × (transverse size) × (transverse size) × 4/3π. Tumor disappearance was judged using histochemical images and luminescent images obtained by using an *in vivo* imaging system (IVIS; PerkinElmer). Tumor cure was defined as the disappearance of luminescence from coelenterazine h and no observation of a tumor histologically.

### Temperature-controlled NIR laser irradiation experiments

Since the results obtained by PTT using the NIR absorbent suggested that the peak of temperature of the tumor during NIR irradiation is the most reliable index to predict tumor shrinkage, we conducted animal experiments using a system that can control the temperature of the tumor. In order to keep the temperature constant, we used a NIR laser system for which the irradiation power is modulated by a temperature feedback control circuit that we previously reported^10^. The temperature was set at 40, 41, 42 or 43 °C (n = 5 in each). The irradiation time was set at 333 s.

### Statistical methods

Data are presented as means ± standard deviation (SD). Statistical analyses were performed using Pearson’s correlation coefficient, the two-sample Kolmogorov-Smirnov test or chi square test with Fisher’s exact test when appropriate. Calculations were performed using GraphPad Prism8 software for Windows. A p value of <0.05 was considered statistically significant.

## Supplementary information

Supplementary Information.
