# Supplementary material for: Highly reliable, targeted photothermal cancer therapy combined with thermal dosimetry using a near-infrared absorbent
Source: Sci Rep. 2020 Jun 17;10:9765. doi: 10.1038/s41598-020-66646-x (PMC7299938; doi:10.1038/s41598-020-66646-x)
Supplement: Supplementary file 1 — Supplementary Information. [file 41598_2020_66646_MOESM1_ESM.docx]

**Supplementary Information**

**Highly reliable, targeted photothermal cancer therapy combined with thermal dosimetry using a near-infrared absorbent**

Shinsuke Nomura^1^, Yuji Morimoto^2^, Hironori Tsujimoto^1^, Masashi Arake^2^, Manabu Harada^1^, Daizoh Saitoh^3^, Isao Hara^4^, Eiichi Ozeki^4^, Ayano Satoh^5^, Eiji Takayama^6^, Kazuo Hase^1^, Yoji Kishi^1^ and Hideki Ueno^1^

^1^Department of Surgery, National Defense Medical College, Saitama 359-8513, Japan

^2^Department of Physiology, National Defense Medical College, Saitama 359-8513, Japan

^3^Division of Traumatology, National Defense Medical College Research Institute, Saitama 359-8513, Japan

^4^Technology Research Laboratory, Shimadzu Corporation, Kyoto 604-8511, Japan

^5^Graduate School of Interdisciplinary Science and Engineering in Health Systems, Okayama University, Okayama 700-0082, Japan

^6^Department of Oral Biochemistry, Asahi University School of Dentistry, Gifu 501-0296, Japan

Corresponding author

Yuji Morimoto, Department of Physiology, National Defense Medical College, 3-2 Namiki, Tokorozawa, Saitama 359-8513, Japan. Tel: +81-4-2995-1483; Fax: +81-4-2996-5188;

E-mail: moyan@ndmc.ac.jp

1. **Effect of heat on the viability of cancer cells (*in vitro* experiment)**

An *in vitro* experiment was performed for confirming the effect of heat on the viability of cancer cells. Five thousand cells/well of NLC26 cells were seeded onto a 96-well plate and incubated for 1 day. The plate was then heated at 39, 40, 41, 42 or 43°C (n = 5, respectively) for 5 min on a silicon rubber heater of which the temperature was controlled by a thermoregulating apparatus (ThreeHigh Co. Yokohama, Japan). The temperature of the cell medium was measured using a thermocouple (ThreeHigh Co. Yokohama, Japan). After 2 days, 10 μl of Cell Counting Kit-8 reagent (Dojindo) was added to each well (containing 100 μl medium) and further incubated at 37°C for 2 h. The viable cells were analyzed using an absorbance photometer (SpectraMax 340PC384. Molecular Devices, LLC. CA, USA) at 450 nm at room temperature (25°C).

The cell viability at 43°C was significantly lower than that at other temperatures (p < 0.0001), (Fig. S1) though there were no differences in cell viability between groups in which the temperature settings were below 42°C. These results suggest that cancer cells are vulnerable to heating at a temperature of 43°C or higher.

**Fig. S1**. Effect of heat on viability of cancer cells (NLC26). The rate of cell viability was calculated by the following formula: rate of cell viability = [number of surviving cells after exposure to heat / number of surviving cells after no exposure to heat]. *p < 0.0001 43°C vs. other groups. Multiple comparisons between groups were made by the Tukey-Kramer test.

1. **Accumulation of ICG lactosome in tumors**

To determine whether ICG lactosome accumulated specifically in tumor tissue, the consistency between the fluorescence from ICG lactosome and the actual tumor location was confirmed using a Colon26 cell line transfected with Nano-lantern luminescent protein (NLC26), which degrades the luminescent substrate coelenterazine h (Wako Pure Chemical Industries Ltd., Osaka, Japan). ICG lactosome (8.8 mg/kg) corresponding to 281 µM of ICG concentration in blood was administered intravenously to the tumor-bearing mice (body weight: approx. 20 g) 48 h before imaging. For visualization of luminescence from the tumor cells, 100 μl of coelenterazine h (Wako Pure Chemical Industries Ltd., Osaka, Japan) solution (2.5 mg/mL) was administered intravenously through the retro-orbital sinus of an anesthetized mouse.

Fluorescent images (ex. 780 nm / em. 845 nm) and luminescence images were obtained using an *in vivo* imaging system (IVIS; Perkin Elmer, MA, US).

Fig. S2A shows a good consistency among the bioluminescent images emitted from a luminescent substrate (coelenterazine h) degraded in tumor cells (BLI) and the fluorescent images from ICG lactosome (FLI). Since fluorescence from ICG lactosome being consistent with luminescence from the tumor was observed, ICG lactosome was revealed to have the property of selective accumulation in the tumor.

Fig. S2B shows time-lapse fluorescent images after the administration of ICG lactosome. At 12 h after administration, the margin of the tumor started to become clear. After that, the tumor/non-tumor tissue fluorescence intensity ratio increased, and the ratio was more than 12-times higher at 48 h after administration.


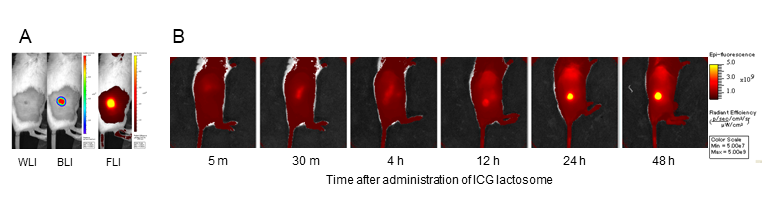


**Fig. S2.** (**A**) Representative examples of a white light image (WLI), bioluminescence image (BLI), and ICG lactosome fluorescent image (FLI) taken at 48 h after intravenous injection of ICG lactosome by IVIS. (**B**) Time-lapse image of fluorescence from ICG lactosome. The location of fluorescence being consistent with the tumor location was identified 12 h after injection of ICG lactosome, and fluorescence at the tumor gradually intensified with time: the contrast ratio (= fluorescence at tumor site /fluorescence at non-tumor site) reached more than twelve at 48 h after administration.

1. **Histological findings of the tumors of mice administered ICG lactosome**

For identifying the distribution of ICG lactosome inside the tumor, hematoxylin-eosin (H&E) and fluorescent images of the tumor were obtained. The tumors were resected 48 hours after the intravenous administration of ICG lactosome (8.8 mg/kg). The resected tumors were mounted in O.C.T. embedding compound and frozen at -20°C. After serial sectioning of the sample, one section was used for fluorescent observation and the other section was stained with H&E. Fluorescent images (ex. 740 nm / em. > 780 nm) were obtained by a fluorescent microscope (Keyence, Tokyo, Japan).

The fluorescence from ICG lactosome was distributed heterogeneously inside the tumor (Fig. S3). Judging from the fluorescent image, it is difficult to determine whether ICG lactosome exists inside the tumor cells or outside the tumor cells; however, ICG lactosome may be outside the tumor cells (be left in the stroma) since internalization of ICG lactosome into tumor cells was not seen (described below, SI.4).


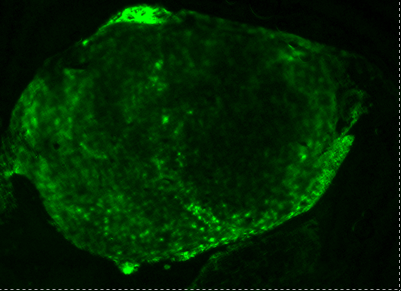


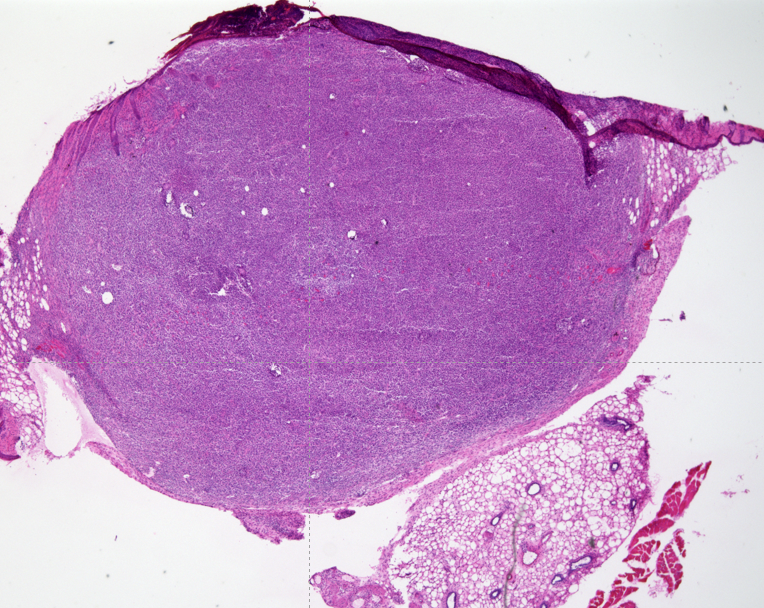


Fluorescent image H&E image

**Fig. S3**. Representative microscopic images of fluorescence and H&E staining of a tumor of a mouse 48 h after injection of ICG lactosome. Scale bar = 1 mm.

1. **Internalization of ICG lactosome into cancer cells is not seen.**

Internalization of ICG lactosome into cancer cells was studied. Cancer cells (Colon26 cells) were incubated with ICG lactosome (1.87 µM) or ICG (1.87 µM) for 1 day. The cells were washed, and the medium was replaced with Hanks' balanced salt solution (HBSS). Fluorescent imaging (ex. 740 nm / em. > 780 nm) was carried out by using a fluorescent microscope.

No cellular uptake of ICG lactosome was observed (Fig. S4. A), though ICG was incorporated into the cytoplasm (Fig. S4. B). Taken together with the results shown in Fig. S3, it was speculated that ICG lactosome neither adheres to the membrane of cancer cells nor is incorporated into cancer cells but accumulates in the stroma of tumor tissue.

**
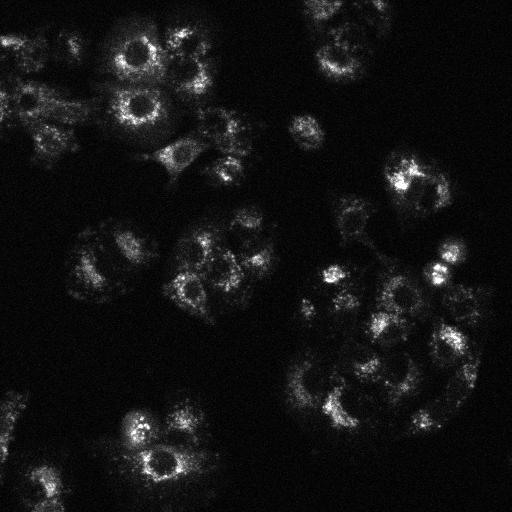
A B**


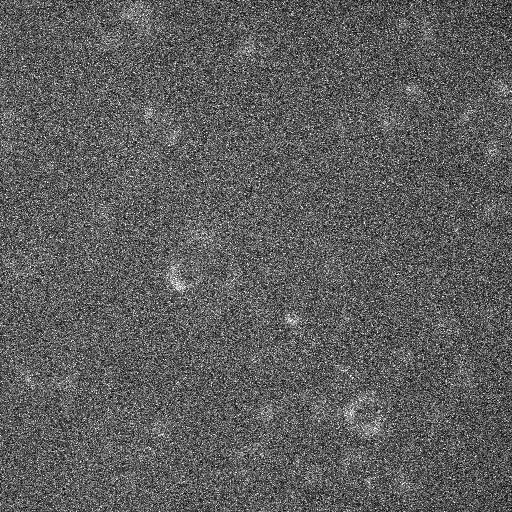


**Fig. S4.** Fluorescent images of Colon 26 cells that were incubated with ICG lactosome (A) or ICG (B) for 24 hours.

1. **Synthesis of ICG-PLLA block copolymer**

**
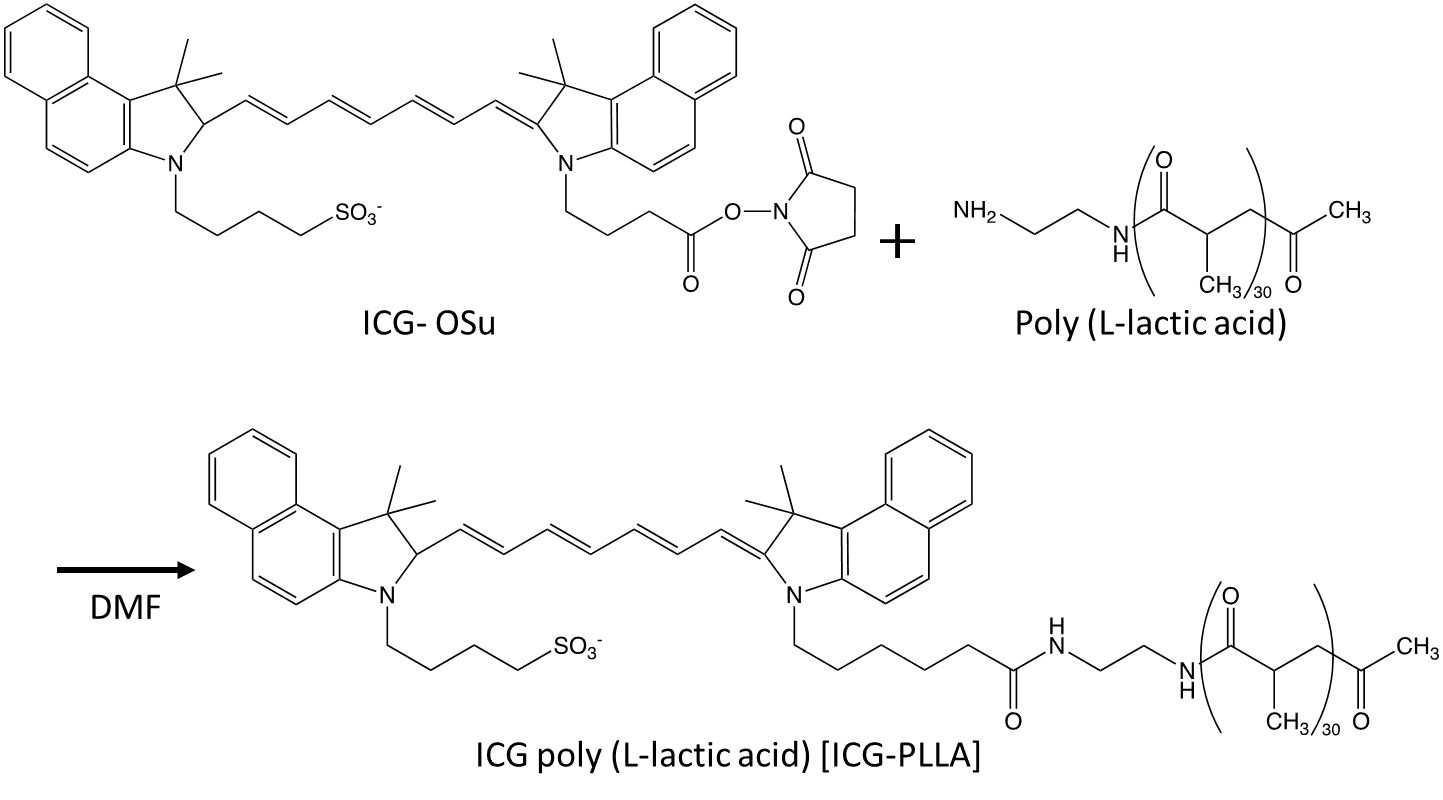
**

**Fig. S5.** Chemical structure of ICG-OSu and the synthesis of ICG-PLLA block copolymer.

1. **Particle properties of ICG lactosomes are thought to be identical to those of lactosomes.**

For discussing the particle properties of ICG lactosomes, the particle properties of lactosomes, which are the basic structure of ICG lactosomes, are first described.

Lactosome is a polymeric micelle (Fig. S6. A) that is an aggregate of amphiphilic polymers. Amphiphilic polymer (PS-PLLA) is composed of poly (sarcosine) [PS: hydrophilic chain] and poly (L-lactic acid) [PLLA: hydrophobic chain]. The static light scattering (SLS) method using a Nano-ZS (Malvern, UK) revealed that one lactosome was composed of 2.7 ± 0.8 × 10^2^ molecules of PS-PLLA.

The dispersion property and particle size of lactosomes were examined by electron microscopy. A drop of dispersion containing lactosomes (2 µL) was mounted on a carbon-coated Cu grid and stained negatively with 2% phosphotungstic acid. Images were obtained using a transmission electron microscope (JEM-2000EXII, JEOL, Tokyo) at an accelerating voltage of 100 kV.

As shown in Fig. S6B, the particles of lactosome were dispersed almost uniformly. Negative staining to clarify the outline of the particles (Fig. S6. C) revealed that the particle size of the lactosome was approximately 30 nm.

The hydrodynamic diameter of lactosome was also determined by the dynamic light scattering (DLS) method. A solution containing lactosomes (1 mg/ml）was filtered by a 0.20-µm polyvinylidene fluoride syringe filter (GE Healthcare UK Limited) and DLS was measured using Nano-ZS.

As shown by the black line in Fig. S6D, particles of lactosomes were distributed over a narrow range (polydispersity index (PDI): 0.126) and the peak of the size distribution was seen at 30.9 nm. Combined with the results obtained by electron microscopic observation, lactosome is considered to have a particle size of approximately 30 nm and the particles are almost uniformly dispersed.

On the other hand, the structure of the ICG lactosome was prefigured to be almost the same as that of the lactosome because most of the composition (80%) of ICG lactosome is PS-PLLA and only 20% of the composition is ICG-PLLA (Fig. S5).

Hence, the particle size distribution of ICG lactosomes was also measured by the DLS method. As a result, the particle size and PDI were revealed to be 31.6 nm and 0.089, respectively (Fig. S6. D, red line). Those values were almost identical to those of lactosomes.

Based on these results, particle properties of ICG lactosomes were considered to be identical to that of lactosomes.

**
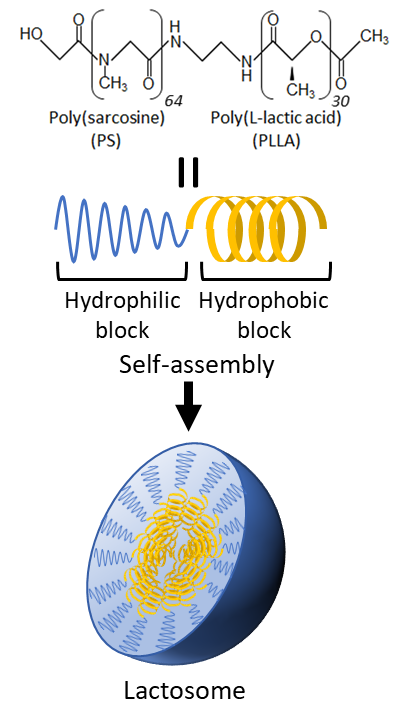
A**

B C


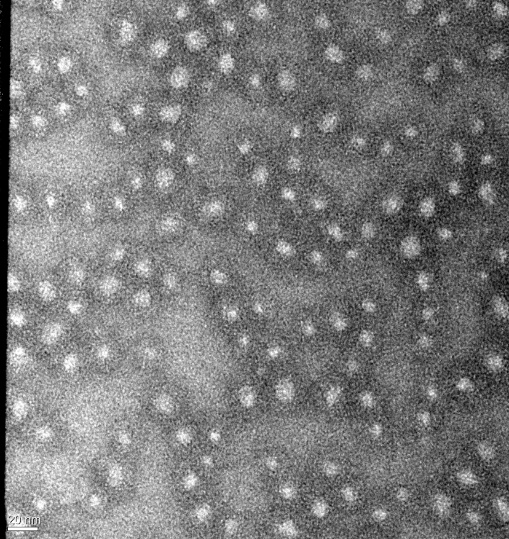

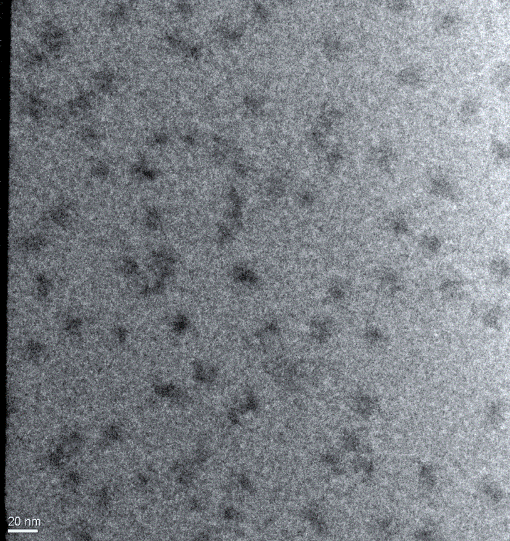


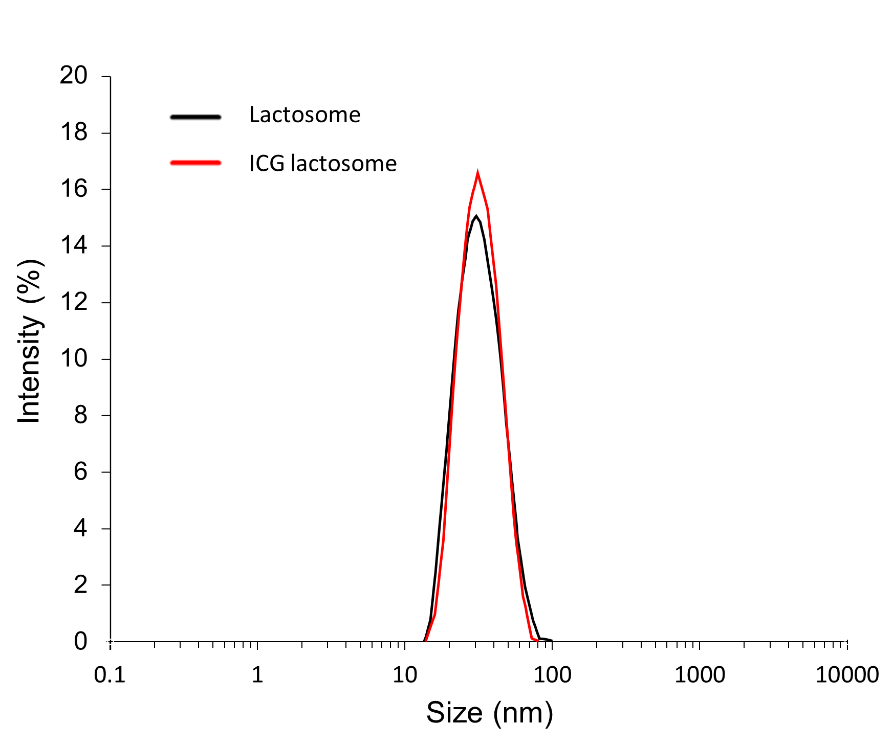
**D**

**Fig. S6**. Chemical structure and illustration of lactosome (A). Transmission electron microscopy images of lactosomes without staining (B) and those of lactosomes stained with 2% phosphotungstic acid (C). Scale bar = 20 nm. Size distributions of lactosomes and ICG lactosomes in distilled water at room temperature, measured by the DLS method (D).

1. **Detailed characterization of ICG lactosome**
2. **Optical properties of ICG lactosome**

For assessing the optical properties of ICG lactosome, absorbance spectra and fluorescence spectra were measured with a spectrometer. Fig. S7A shows absorbance and fluorescence curves of ICG lactosome dissolved in distilled water (10 µM) and Fig. S7B showed those of ICG dissolved in distilled water (15 µM). The maximum absorbance of ICG lactosome is 796 nm and that of ICG is 778 nm. The fluorescence spectra of ICG lactosome and ICG showed maximum emissions at 815 nm and 807 nm, respectively, when excited at 695 nm.

The red shifts seen in both the absorbance spectra and fluorescence spectra of ICG lactosome correspond to the changes in the physicochemical environment and are consistent with a specific interaction of the ICG molecules^1,2^.


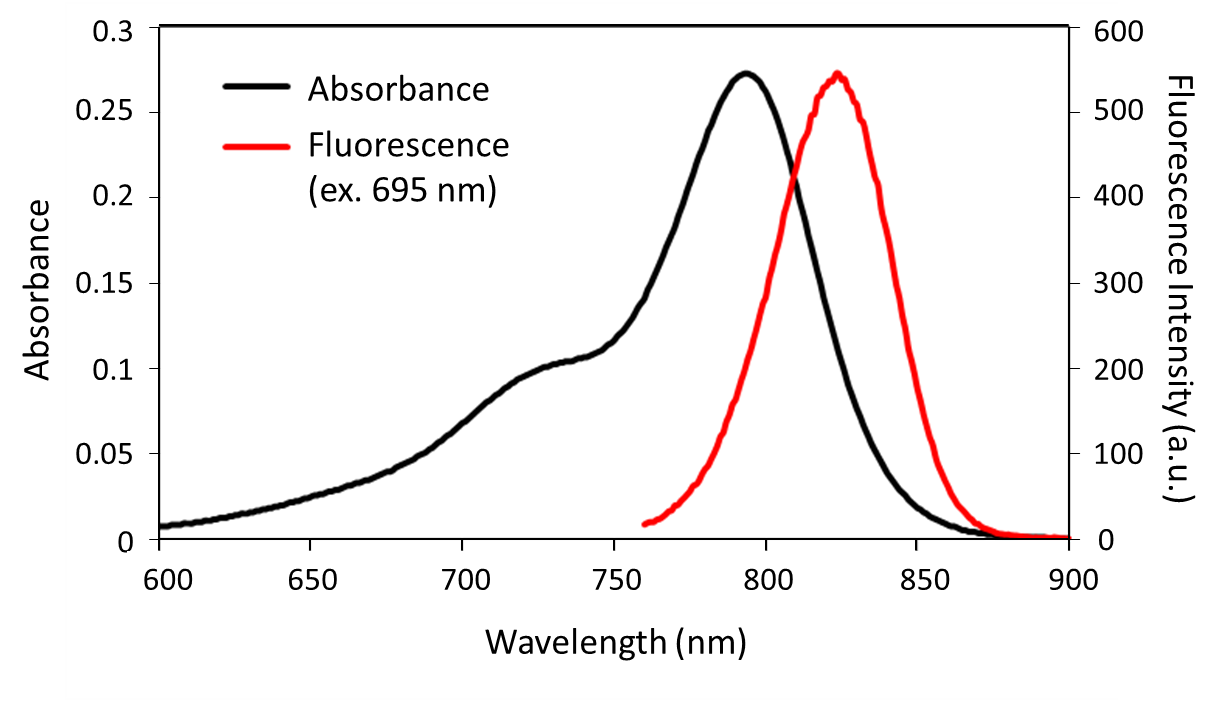
**A**

**B**

**
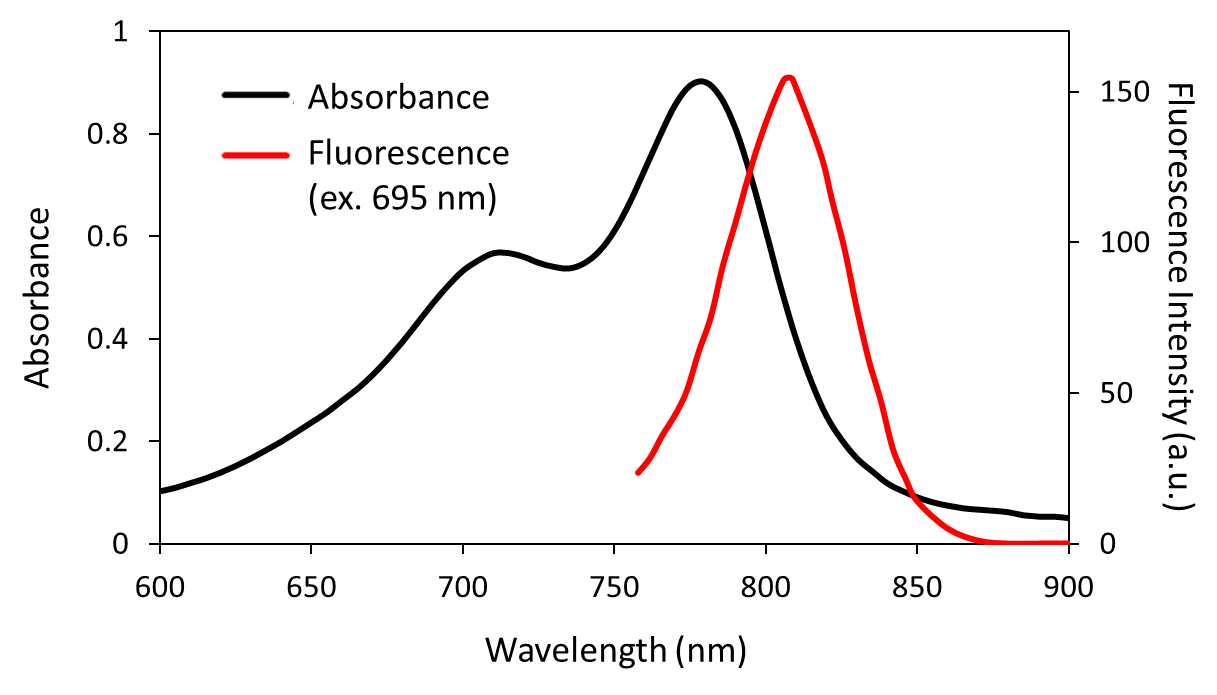
**

**Fig. S7.** Absorbance and fluorescence spectra of ICG lactosome (A) and ICG (B).

1. **Degradation of ICG lactosome**

The temporal degradation of ICG lactosome was confirmed by measuring the size distribution using a DLS system. When ICG lactosome dissolved in distilled water was stored under a light shield at room temperature for 21 days, particles of more than 100 nm in size appeared on day 3 or later (Fig. S8. A), suggesting that aggregation began on day 3 at room temperature. However, in the case of ICG lactosome stored at 4°C (under a light shield), aggregation was not observed until 21 days after the start of storage (Fig. S8. B), suggesting that the dispersion stability of ICG lactosome at 4°C is much greater than that at room temperature.

Next, the temporal optical stability of ICG lactosome was compared to that of ICG: temporal changes in absorbance spectra of ICG lactosome in distilled water and ICG in distilled water when both were stored at 4°C under a light shield were acquired with a spectrometer. The absorbance spectra of ICG lactosome did not change over a storage period of 64 days (Fig. S8. C); however, those of ICG changed: the peak absorbance of ICG started to decrease even 1 day after the storage and continued to decrease with increase in storage time (Fig. S8. D). The results show that the optical stability of ICG lactosome is much greater than that of ICG.

**
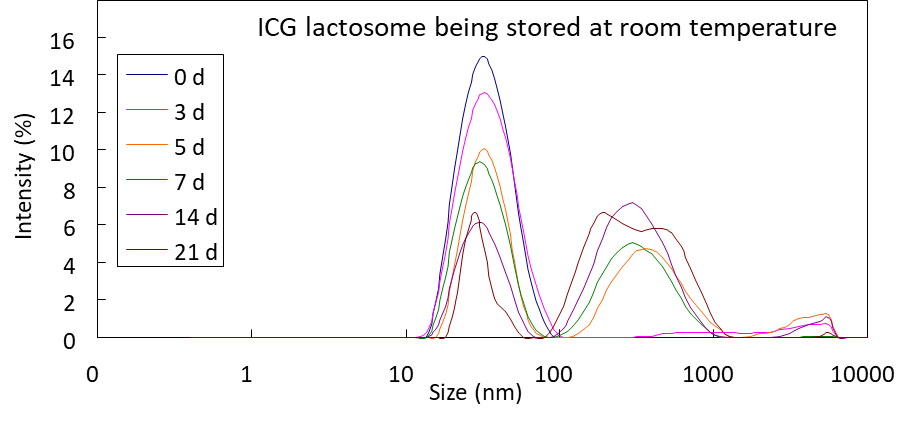
A**

**B**


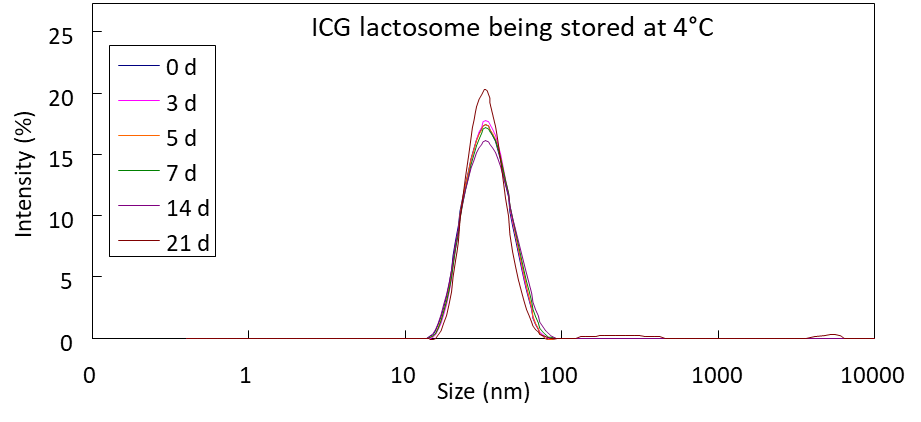


0

5

10

15

20

25

0

1

10

100

1000

10000

Size (nm)

Intensity (%)

0d

3d

5d

7d

2w

3w

**
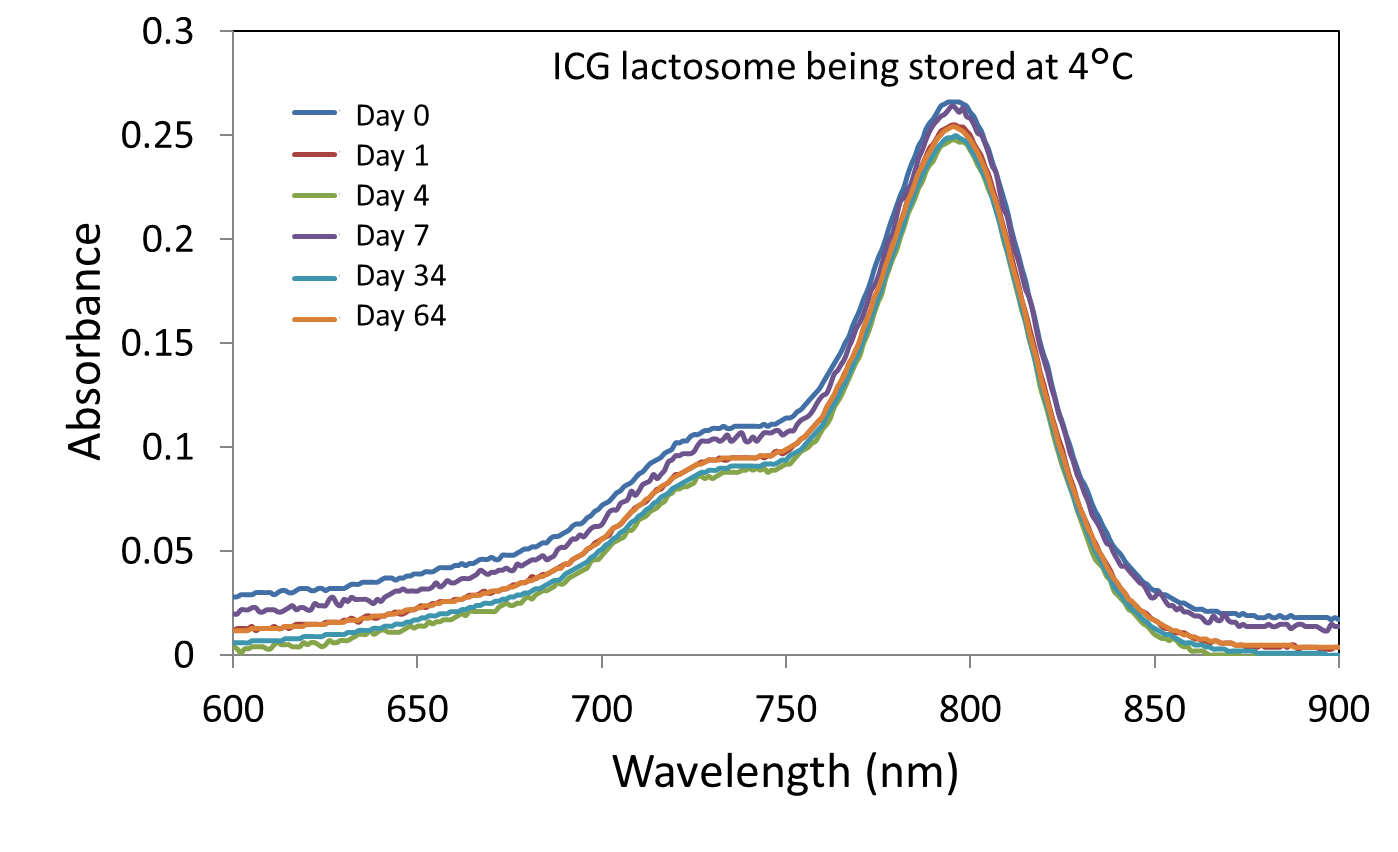
C**

**
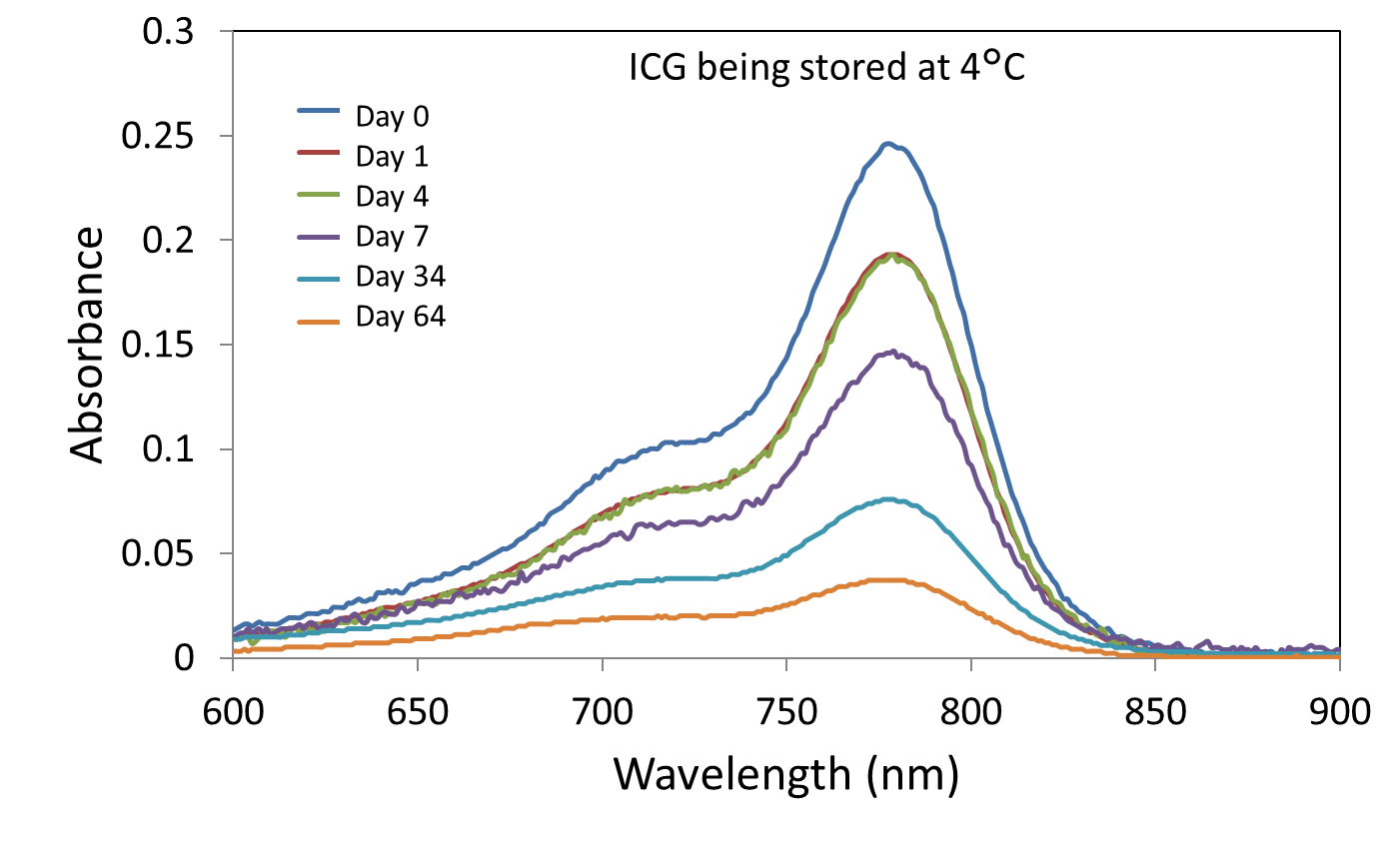
D**

**Fig. S8.** Temporal changes in the size distribution of ICG lactosome in distilled water at room temperature (A) and those of ICG lactosome in distilled water at 4°C (B). Temporal changes in absorbance of ICG lactosome in distilled water at 4°C (C) and those of ICG in distilled water at 4°C (D).

1. **Fluorescent property of ICG lactosome**

For the purpose of studying the fluorescent property of ICG lactosome, the intensities of fluorescence from ICG lactosome and ICG were compared.

The fluorescence intensities of ICG lactosome and ICG in distilled water and in albumin (8.2 μM)-water solution were measured. The intensity of fluorescence (ex. 740 nm / em. > 780 nm) from ICG lactosome (281 μM) was about three-times higher than that of ICG (281 μM) in both distilled water and albumin-water solution^3^ (Fig. S9). The results suggest that the quantum yield of ICG lactosome is greater than that of ICG.


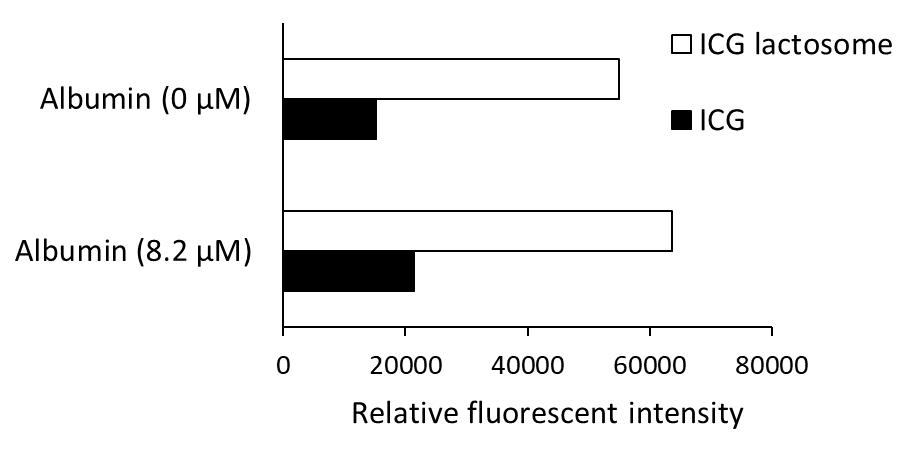


**Fig. S9.** Relative fluorescent intensities of ICG lactosome and ICG^3^.

1. **Acute toxicity effect of lactosome**

To confirm the acute toxicity effect of lactosome, Balb/c mice (n = 3, each sex) at 7 weeks of age (Japan SLC, Hamamatsu, Japan) were used and lactosome (2000 mg/kg) was systemically administered.

No changes in body weight or anatomical findings were observed during a period of 11days after administration.

**Supplementary References**

1. Bae, P. K., Jung, J. & Chung, B. H. Highly enhanced optical properties of indocyanine green/perfluorocarbon nanoemulsions for efficient lymph node mapping using near-infrared and magnetic resonance imaging. *Nano convergence*. **1**, 1-10 (2014).

2. Mordon, S., Devoisselle, J. M., Soulie-Begu, S. & Desmettre, T. Indocyanine green: physicochemical factors affecting its fluorescencein vivo. *Microvasc. Res.* **55**, 146-152 (1998).

3. Tsujimoto, H. *et al.* Photodynamic therapy using nanoparticle loaded with indocyanine green for experimental peritoneal dissemination of gastric cancer. *Cancer Sci.* **105**, 1626-1630 (2014).
